# The formation mechanism of Li_4_Ti_5_O_12−*y*_ solid solutions prepared by carbothermal reduction and the effect of Ti^3+^ on electrochemical performance

**DOI:** 10.1038/s41598-019-41206-0

**Published:** 2019-03-18

**Authors:** Guijun Yang, Soo-Jin Park

**Affiliations:** Department of Chemistry, Inha University, 100 Inharo, Incheon, Korea

## Abstract

Samples of Li_4_Ti_5_O_12−*y*_ solid solutions are synthesized by one-step solid-state carbothermal reduction reaction using Li_2_CO_3_, anatase, and carbon black under a nitrogen atmosphere. The underlying formation mechanism that leads to Li_4_Ti_5_O_12−*y*_ solid solutions is proposed. The formation mechanism of the Li_4_Ti_5_O_12−y_ solid solution is investigated by *in situ* variable temperature X-Ray diffraction (VT-XRD) and thermogravimetric analysis/differential scanning calorimetry (TGA-DSC). First, some Ti^4+^ centers are converted to Ti^3+^ (TiO_2_-TiO_2−*x*_) because of the presence of carbon black. Secondly, Li_2_CO_3_ reacts with TiO_2−*x*_ (anatase) to form Li_2_TiO_3_. Thirdly, Li_2_TiO_3_ reacts with TiO_2−*x*_ to form the Li_4_Ti_5_O_12−*y*_ solid solution, while anatase starts to transform into rutile at the same time. Rutile reacts with Li_2_TiO_3_ to form Li_4_Ti_5_O_12−*y*_ at higher temperatures. The presence of Ti^3+^ not only improves the electrical conductivity but also improves the ionic conductivity. As a result, the as-prepared material exhibits good rate capability and cycling stability with 99.3% capacity retention after 200 cycles.

## Introduction

Spinel Li_4_Ti_5_O_12_ (LTO) has already been employed as anode material in many fields, such as hybrid electric vehicles (HEVs), plug-in hybrid vehicles (PHEVs), and electric vehicles (EVs)^1–4^. As an alternative to carbon anodes, Li_4_Ti_5_O_12_ has a long flat charge/discharge voltage platform (1.55 V vs. Li/Li^+^), which is higher than the reduction potentials of most electrolyte solutions, thus avoiding the formation of solid-electrolyte interphase (SEI) layer (which usually occurs below 1.0 V vs. Li/Li^+^) and improving safety^5–7^. LTO is known as a “zero strain material” because the change of Li_4_Ti_5_O_12_ volume is small during the lithium-ion insertion and extraction process, which results in a long-term cycling life^8–10^. Furthermore, a high coulombic efficiency, long cycling performance, excellent safety, and a relatively high reversible capacity of 175 mA h g^−1^ make Li_4_Ti_5_O_12_ a promising anode material. However, the empty Ti 3d state with a band gap of ca. 2 eV still results in poor rate capability^11–14^. So far, considerable effort has been made to improve the reaction kinetics of LTO. Among these methods, the use of a conductive surface coating is the most common method. Carbon-based coatings are the most widely used type, including glucose^15^, sucrose^16^ soybean oil^17^, pitch^18^, poly(3,4-ethylenedioxythiophene) (PEDOT)^19^, and citric acid^20^. The organic carbon source undergoes a series of chemical reactions with increasing temperature in the carbonization process. The H, O, and N fraction in the carbon source decrease, the carbon content is continuously enriched, and pure carbon is formed in the end. A conductive coating can improve the surface conductivity, which can directly affect the electrochemical performance of materials. Wang *et al*.^21^ hypothesized that the carbonization not only provides a carbon coating layer but also partially reduces Ti^4+^ to Ti^3+^ at the contact surface of carbon and LTO. The mixed Ti^4+^/Ti^3+^ can improve the electrochemical performance of materials. Thus, a proper surface modification is a promising way to improve the electrochemical behavior of materials.

Li_4_Ti_5_O_12_ is a composite oxide of the AB_2_X_4_ series, such as LiMn_2_O_4_. LTO is a part of the spinel solid solution Li_1+*x*_Ti_2−*x*_O_4_ (0 ≤ *x* ≤ 1/3). According to the structure of Li_4_Ti_5_O_12_, the formula can be described as Li_8a_ [Li_1/3_Ti_5/3_]_16d_ [O_4_]_32e_. The subscripts indicate the number of equivalent sites with Wyckoff symbols for the space group *Fd*-3*m*^22^. During the Li^+^-ion insertion/extraction process of Li_4_Ti_5_O_12_, the chemical composition and electrochemical properties of the crystal lattice change continuously with Li^+^-ion migration. When Li^+^ ions are inserted to the structure of spinel Li_4_Ti_5_O_12_, the foreign Li^+^ ions occupy the octahedral 16c sites of the lattice, which are close to the tetrahedral 8a sites, and the original lithium ions of the 8a sites also migrate to the octahedral 16c sites^23^. Finally, all the 16c sites are occupied by lithium ions and a rock salt phase of [Li_2_]_16c_ [Li_1/3_Ti_5/3_]_16d_ [O_4_]_32e_ is formed, which results in a transition from insulator Li_4_Ti_5_O_12_ to Li_7_Ti_5_O_12_ with good conductivity with increasing lithium-ion content in the lattice^24^. Li_7_Ti_5_O_12_ has a good electrical conductivity (10^−2^ S cm^−1^), higher than that of Li_4_Ti_5_O_12_ (10^−1^3 S cm^−1^). The most important factor is that the spinel host structure accommodates lithium ions without a significant change to the lattice constant^25,26^. This is an interesting phenomenon and has attracted much attention. Wangemaker *et al*.^27^ reported that the use of Li_4_Ti_5_O_12−*y*_ solid solutions could improve the Li^+^-ion mobility, which can speed up the charge/discharge process. Recently, Qiu *et al*.^28^ prepared blue-colored hydrogenated Li_4_Ti_5_O_12_ by treating commercial Li_4_Ti_5_O_12_ at 500 °C under a 40 bar hydrogen atmosphere. Li_4_Ti_5_O_12_ exhibits a high discharge capacity and superior rate capability. Nie *et al*.^29^ prepared Ti^3+^ self-doped Li_4_Ti_5_O_12_ nanosheets via a facile solvothermal approach combined with a hydrogenation treatment. The Li_4_Ti_5_O_12_ nanosheets exhibit excellent rate capability and good cycling stability. In addition, spinel LiTi_2_O_4_, which belongs to the AB_2_X_4_ series, has a significantly higher electrical conductivity of 5.56 × 10^6^ S cm^−1^ compared to Li_4_Ti_5_O_12_^30^. However, they concluded that the presence of Ti^3+^ has a great effect on the electrical conductivity of Li_4_Ti_5_O_12_. The formation mechanism of Ti^3+^ and the relationship between Ti^3+^ and electrochemical performance of materials were not studied in detail.

In this study, we prepared Li_4_Ti_5_O_12−*y*_ solid solutions using carbon black, and the formation mechanism of Li_4_Ti_5_O_12_ in the presence of carbon (carbon black) was investigated. Moreover, the effect of Ti^3+^ on the electrochemical performance of the Li_4_Ti_5_O_12−y_ solid solutions has been investigated systematically. The electrochemical performance of the prepared Li_4_Ti_5_O_12−*y*_ solid solution was also studied.

## Experimental

### Material Preparation

Samples of Li_4_Ti_5_O_12−*y*_ solid solutions were synthesized by a one-step carbon thermal reduction method. Stoichiometric amounts of Li_2_CO_3_ and TiO_2_ (anatase) were used as lithium and titanium sources, respectively. A 5% excess of Li_2_CO_3_ was added to the reactants because of the ease of evaporation of the lithium source at high temperatures. Carbon black was not used as the carbon source but as a reductant. Typically, 0.79 g Li_2_CO_3_, 2.02 g TiO_2_, and different content of carbon black were thoroughly mixed in agate mortar, and then heated at 850 °C in a tube furnace under a nitrogen atmosphere for 10 h. The carbon black loadings used were 0, 1, 2, and 3 wt.%, of the total weight of Li_2_CO_3_ and TiO_2_, and the obtained materials were labeled as P-LTO, 1-LTO, 2-LTO, and 3-LTO, respectively.

### Material Characterization

*In situ* variable temperature X-Ray diffraction (VT-XRD) was carried out on a PANalytical X’Pert powder diffractometer with Cu Kα radiation (λ = 1.5405 Å), equipped with an Anton Parr HTK 1200 N high-temperature heating apparatus for the identification of phases and phase transitions. For the *in situ* high temperature VT-XRD study, the Li_4_Ti_5_O_12_ mixture was pressed into a square tablet with an area of 100 mm^2^ and thickness of 2 mm. Then, the tablet was heated in a N_2_ atmosphere from room temperature to 600 °C at a heating rate of 10 °C min^−1^, and the first XRD pattern was collected. Then, the sample was heated to 900 °C, pausing for 30 min at every 50 °C interval to collect the XRD data from 20 to 50° (2θ) (Fig. SI 1). The XRD patterns are labeled to distinguish the patterns collected at each stage during heating process. For example, the XRD pattern at 600 °C is denoted 600-1 and the pattern kept for 30 min is denoted 600-2, respectively. Thermaogravimetric analysis (TGA-DSC) was conducted on a STA409C/PCPFEIFFER VACUUM TGA-7 analyzer (NETZSCH-Ger tebau GmbH, Germany) in a nitrogen atmosphere with a flow rate of 30 mL min^−1^ from 40 to 900 °C at a heating rate of 3 °C min^−1^. The phase identification was performed by XRD (Panalytical Incorporated, Netherlands) from 15° to 50°. The morphologies of the samples were obtained using high-resolution scanning electron microscopy (HR-SEM, SU 8010, Hitachi, Ltd., Japan). UV-Vis diffuse reflectance spectra of the samples were recorded over the range of 200–800 nm in absorption mode using a Thermo Nicolet Evolution 500 UV-Vis spectrophotometer equipped with an integrating sphere attachment. The X-ray photoelectron (XPS) spectra were obtained with ESCALAB250 XPS (Thermo Fisher Scientific, USA) at 2 × 10^−9^ mbar. Al Ka (1486.6 eV) was used as the X-ray source at a 15-keV anode voltage, and all binding energies were referenced to the C1s peak (284.8 eV) arising from adventitious carbon.

### Electrochemical Measurements

Coin cells (CR2025) were assembled to test the electrochemical performance of the obtained samples for comparison. The working electrode was prepared by coating a slurry of active material (80 wt.%), carbon black (electronic conductive additive, 10 wt.%), and polyvinylidene fluoride (PVDF, binder, 10 wt.%) in *N-*methyl pyrrolidone (NMP) solution onto a copper foil. The obtained electrodes were then dried at 110 °C overnight under vacuum. The half cells were assembled in an Ar-filled glove box. Celgard 2400 and lithium were used as the separator and counter electrode, respectively. 1 M LiPF_6_ in ethylene carbonate (EC), dimethyl carbonate (DMC), and ethylene methyl carbonate (EMC) (1: 1: 1 by volume) was used as electrolyte. The galvanostatic charge/discharge characteristics of the cells were analyzed in a voltage range of 1.0–2.5 V (vs. Li/Li^+^) at various rates using a LAND CT2001 battery tester. The specific capacity was calculated based on the active mass of the electrode.

Cyclic voltammetry (CV) curves were performed on a Zennium/IM6 electrochemical workstation (Zahner, Germany) in the voltage range of 1.0–2.5 V (vs. Li/Li^+^) at different scan rates. Electrochemical impedance spectroscopy (EIS) measurements were obtained on the same workstation with an oscillating voltage of 5 mV over frequency ranging from 10^−2^ to 10^5^ Hz.

## Results and Discussion

**Figure 1:**
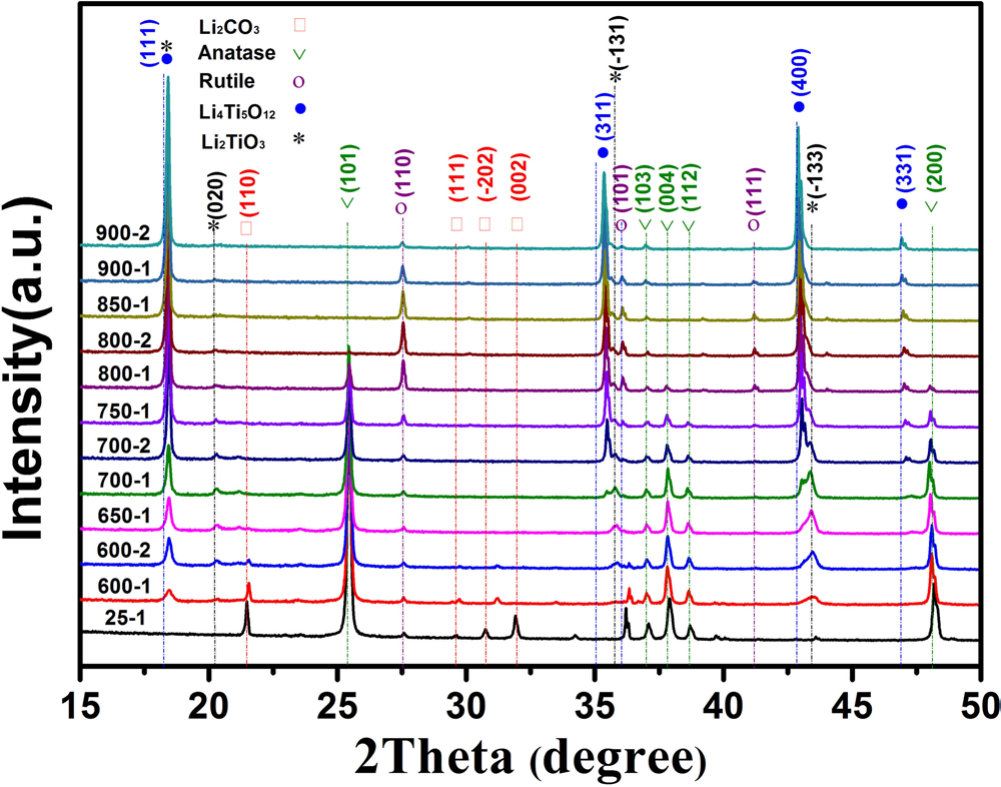
The *in situ* VT-XRD patterns of the mixture (Li_2_CO_3_, anatase, and carbon black) from room temperature to 900 °C.

The *in situ* VT-XRD measurements were used to analyze the formation mechanism of LiTi_4_O_5_ during the carbothermal solid-state reduction reaction. The *in situ* VT-XRD patterns obtained on heating the sample from room temperature to 900 °C in a 2*θ* range of 15–50° are shown in Fig. 1. The *in situ* VT-XRD patterns of the mixture (25-1, Li_2_CO_3_, anatase, and carbon black) before heating are consistent with the corresponding crystal structure in the inorganic database (ICSD). No peaks of carbon black were observed because it is amorphous, and there is little rutile in the reactant mixture. Figure 1 shows that the intensity of Li_2_CO_3_ (JCPDS CARD No. 01-072-1216) and anatase (JCPDS CARD No. 00-002-0387) decreased significantly at 600 °C. The peaks at 20.17°, 35.8° and 43.5° which correspond to the (0 2 0), (-1 3 1) and (-1 3 3) planes of intermediate Li_2_TiO_3_ (JCPDS CARD No. 00-033-0831) appeared. The intensity of peaks corresponding to Li_2_TiO_3_ increased with the decrease in the Li_2_CO_3_ reactant until Li_2_CO_3_ was consumed at 600-2. The peaks at 35.5°, 43.2°, and 47.3° corresponding to the (3 1 1), (4 0 0), and (3 3 1) planes of Li_4_Ti_5_O_12_ (JCPDS CARD No. 00-049-0207) were observed at 700 °C, which implies that Li_4_Ti_5_O_12_ product was generated from 700 °C (700-1). In fact, the two peaks at 18° corresponding to (1 1 1) planes of Li_4_Ti_5_O_12_ and Li_2_TiO_3_ were not distinguishable because of the overlapping of these two phases. After heating to 800 °C for 30 min, the anatase peaks disappeared completely, and the intensity of the peaks of the rutile phase (JCPDS CARD No. 01-086-0147) increased rapidly at the same time (800-1). The increased intensity of rutile peaks is due to the transformation of anatase to rutile. In fact, anatase began to turn into rutile at 700 °C (700-1), but the phase transition process occurred rapidly at 800 °C. After anatase disappeared, the peak intensity corresponding to rutile decreased at 850 °C (850-1), together with the decrease of Li_2_TiO_3_. Rutile is more stable than anatase and it requires more time and a higher temperature to react with Li_2_TiO_3_. This explains why researchers often use anatase.

**Figure 2:**
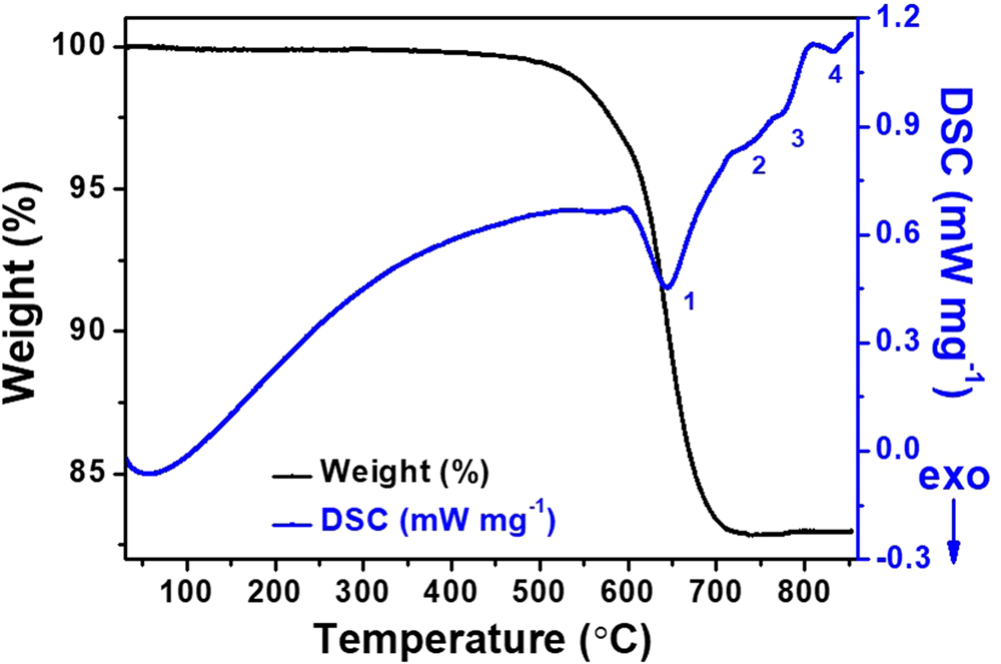
TGA-DSC curves of mixed reactant measured from room temperature to 900 °C.

To confirm the formation mechanism of Li_4_Ti_5_O_12_ during the solid state reaction further, the reactant mixture (Li_2_CO_3_, anatase, and carbon black) was examined by TGA-DSC from room temperature to 900 °C (Fig. 2). Under hypoxic conditions, to maintain electrical neutrality, the reduction of Ti^4+^ to Ti^3+^ is accompanied by the formation of oxygen vacancies^31^.

1 $$2Ti{O}_{2}(anatase)+\frac{1}{2}C\mathop{\longrightarrow }\limits^{ < {600}^{\circ }{\rm{C}}}2T{i^{\prime} }_{Ti}+{V}_{O}^{\bullet \bullet }+3{O}_{O}+\frac{1}{2}C{O}_{2}(Ti{O}_{2-x})$$

It is worth noting that Chen *et al*.^32^ reported oxygen-deficient Li_4_Ti_5_O_12−y_ by annealing the product under an N_2_ atmosphere at 500 °C. This approach takes advantage of the defective mesoporous structure with oxygen vacancies and Ti^3+^-O-Ti^4+^ pairs to achieve higher electrical conductivity and better electrochemical performance of LTO.

In the TGA curves, the first weight loss is 0.12% at 200 °C, and this is due to the dehydration process of the reactant. The second weight loss is 16.44 wt.% at 450–710 °C, corresponding to a broad exothermic peak at 600–710 °C in the DSC curve, which corresponds well to the theoretical weight loss of CO_2_ from Li_2_CO_3_ (15.6 wt.%). Here, ball milling can decrease the temperature of CO_2_ loss (<400 °C)^33,34^. Therefore, there are different reaction temperatures under different conditions. The reaction equation from 450–710 °C are shown in Eq. (2).

2 $$L{i}_{2}C{O}_{3}+Ti{O}_{2-x}(anatase)\to L{i}_{2}Ti{O}_{3-x}+C{O}_{2}$$

There is no weight loss in the TGA curve from 710–760 °C, but there is a small broad endothermic peak in the DSC curve. This DGA-DSC curve corresponds to the *in situ* VT-XRD patterns in Fig. 1, where intermediate Li_2_TiO_3_ reacts with anatase and carbon black to generate the target product, Li_4_Ti_5_O_12−*y*_.

3 $$2L{i}_{2}Ti{O}_{3-x}+3Ti{O}_{2-x}(anatase)\to L{i}_{4}T{i}_{5}{O}_{12-y}$$

A third tiny exothermic peak from 760–810 °C was observed in the DSC curve with no corresponding weight loss in the TGA curve. This temperature range corresponds to the phase transition of anatase to rutile in the *in situ* VT-XRD patterns (Fig. 1). The equation is shown in Eq. (4).

4 $$Ti{O}_{2-x}(anatase)\to Ti{O}_{2-x}(rutile)$$

The final small exothermic peak appeared between 810–870 °C, but there is no weight loss. According to the *in situ* VT-XRD patterns shown in Fig. 1, the equation for the reaction of Li_2_TiO_3_ with rutile and carbon black to generate the target product Li_4_Ti_5_O_12−*y*_ is given by Eq. (5).

5 $$2L{i}_{2}Ti{O}_{3-x}+3Ti{O}_{2-x}(rutile)\to L{i}_{4}T{i}_{5}{O}_{12-y}$$

There are two exothermic peaks in DSC curve with no weight loss during the cooling process. These peaks are marked as peaks 5 and 6 at the same temperature range as peaks 4 and 3 respectively. This suggests that the two pairs of peaks correspond to the same reaction. No obvious exothermic peak weight loss was observed below 750 °C.

Based on the above results, the formation mechanism of solid solution Li_4_Ti_5_O_12−y_ can be summarized by Eqs (1–5). In addition, the schematic of the formation mechanism is shown in Fig. SI 2.

**Figure 3:**
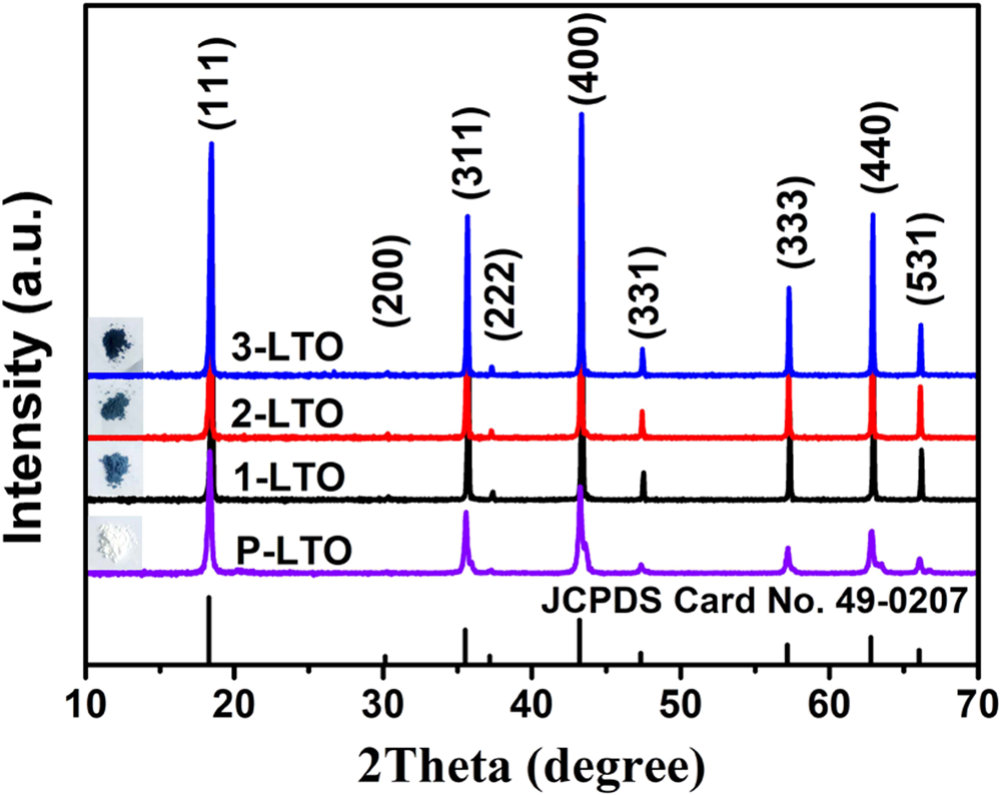
XRD pattern of Li_4_Ti_5_O_12−*y*_ with different carbon black contents.

To investigate the effects of carbon black on the electrochemical performance of LTO, a series of comparative experiments were conducted. Figure 3 presents the XRD patterns of the P-LTO, 1-LTO, 2-LTO, and 3-LTO, respectively. XRD measurements were conducted to examine the crystal structure of the four prepared samples. All samples showed typical diffraction peaks corresponding to the planes of LTO crystals (JCPDS no. 00-049-0207). No diffraction peaks of carbon or impurity phases were detected, suggesting that the presence of carbon black in the formation Li_4_Ti_5_O_12_ did not significantly affect the bulk crystalline structure of the LTO. As demonstrated, the phase structures of the samples are the same. However, the colors of the samples are quite different. P-LTO is white, whereas the others (1-LTO, 2-LTO, and 3-LTO) are blue.

The morphologies of P-LTO and 2-LTO were investigated by SEM. As shown in Fig. SI 3a, the P-LTO particles are irregularly shaped cubes with a size range of about 1–2 μm. Compared with the P-LTO sample, 2-LTO particles (Fig. SI 3b) shows regular cubic geometry and relatively uniform size.

Typically, the color change of LTO during the reaction process is attributed to oxygen vacancies, oxygen deficiency, and surface disorder^35,36^. This can be demonstrated by spectroscopic analysis using UV-vis diffraction spectroscopy, as shown in Fig. SI 4. Both the white and blue LTO absorb UV light, which is determined by the inherent bandgap. The UV-vis spectrum of the P-LTO is characterized by the strong absorption of UV radiation in the range 200–300 nm, and no absorption was observed in the visible light region (400–800 nm). This explains why the P-LTO is white. Visible light absorption can be attributed to the creation of mid-gap states between the conduction band and valence band. The blue samples have similar absorption profiles in the UV region, although these are different from that of P-LTO and exhibit a new and remarkable absorption in the visible region, especially in the red and infrared regions, subsequently yielding the blue color and suggesting the existence of Ti^3+^. Therefore, oxygen-deficient LTO can be regarded as a solid solution of Ti^3+^ and Ti^4+^. The 2-LTO and 3-LTO samples show similar absorption profiles in the visible light region. The 1-LTO sample also showed significant visible light absorption. Calcination with carbon black led to the formation of oxygen vacancies in the LTO lattice. Here, acetylene black served as a reductant to induce the partial reduction of Ti sites (Ti^4+^ to Ti^3+^).

**Figure 4:**
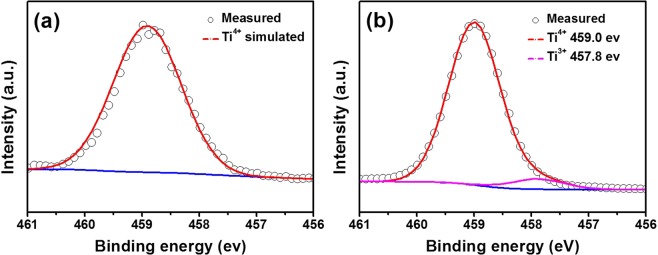
X-Ray photoelectron spectra for Li_4_Ti_5_O_12−*y*_ with different carbon black contents: (**a**) P-LTO and (**b**) 2-LTO.

XPS analysis was carried out to investigate the Ti-ion valence state of the P-LTO and 2-LTO samples further. The XPS data were corrected by taking into account the binding energy of the C1s line of residual carbon at 284.6 eV. As shown in the spectra of P-LTO (Fig. 4a), there are two peaks centered at 458.7 and 457.8 eV, corresponding to the characteristic peaks of Ti^4+^ and Ti^3+^ species. No trivalent Ti was observed in P-LTO. Accordingly, the XPS spectra of Fig. 4b suggest mixed valence Ti^3+^/Ti^4+^ in the 2-LTO sample with a carbon additive. The carbon additive provides a reductive atmosphere and induces the partial reduction of Ti^4+^ to Ti^3+^, which would lead to an increase in number of electrons and improvement in the electrical conductivity. Because of the larger ionic radius of Ti^3+^ (0.67 Å) compared to Ti^4+^ (0.605 Å)^37^, the presence of Ti^3+^ will affect the lattice parameter and make Li^+^-ion migration easier. The higher electrical conductivity results in improved interfacial charge transfer kinetics, which can result in good rate performance.

TGA measurement was carried out from room temperature to 800 °C under air atmosphere to calculate the residual carbon content. As shown in Fig. SI 5, the first weight loss below 200 °C could be attributed to the moisture of the samples. The slight weight rise around 130–230 °C can be attributed to the oxidation of Ti (III). The content of residual carbon can be easily estimated based on the weight loss caused by the carbon oxidation in the range of 300–550 °C. The residual carbon content of 1-LTO, 2-LTO and 3-LTO are 0.33%, 0.78% and 2.48%, respectively. It can be clearly seen that the amount of residual carbon of 3-LTO is seriously excessive. Compared with active material Li_4_Ti_5_O_12_, the carbon additive provides a negligible amount of capacitance during the charge and discharge process, so if there is an excessive amount of carbon additive in the active material, its specific capacitance inevitably decreases.

To investigate the electrochemical kinetics, CV measurements were conducted at a scan rate of 0.5 mV s^−1^ in the voltage range of 1.0–2.5 V (Fig. SI 6). A single pair of oxidation and reduction peaks (around 1.5 and 1.6 V), which corresponds to the Ti^4+^/Ti^3+^ redox couple^38^, was observed for all the samples. The 2-LTO exhibits a pair of obvious oxidation and deoxidization peaks at 1.52 and 1.62 V, corresponding to the charge and discharge plateaus. The P-LTO, 1-LTO, and 3-LTO samples have pairs of oxidation and reduction peaks at 1.51 and 1.65, 1.48 and 1.69, and 1.52 and 1.65 V, respectively. The potential difference (φ_a_-φ_b_) between the anodic and cathodic peaks can reflect the degree of polarization of the electrode. The potential difference of the 2-LTO electrode (0.1 V) is lower than that of others and has a better overlap of the CV curves for 10 cycles, which suggests that 2-LTO electrode has the best reversibility of the redox reaction.

**Figure 5:**
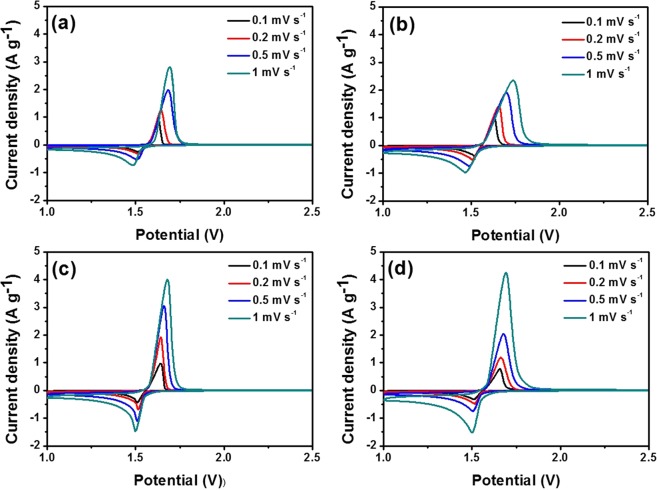
Cyclic voltammetry of (**a**) P-LTO (**b**) 1-LTO (**c**) 2-LTO, and (**d**) 3-LTO at different scan rates (0.1, 0.2, 0.5, and 1 mV s^−1^).

To investigate the influence of Ti^3+^ on the kinetic performance further, the CV curves of the four samples were obtained at different scan rates (0.1, 0.2, 0.5, and 1 mV s^−1^), as shown in Fig. 5. The peak currents and the potential difference increased with increasing scan rate. All the peak current densities of blue LTO (1-LTO, 2-LTO, and 3-LTO) are higher than those of P-LTO; in particular, 2-LTO retains a stable shape redox peaks with increasing scan rate. These results indicate that Ti^3+^ accelerates the lithium-ion insertion and extraction process, particularly affecting the process of lithium-ion insertion.

**Figure 6:**
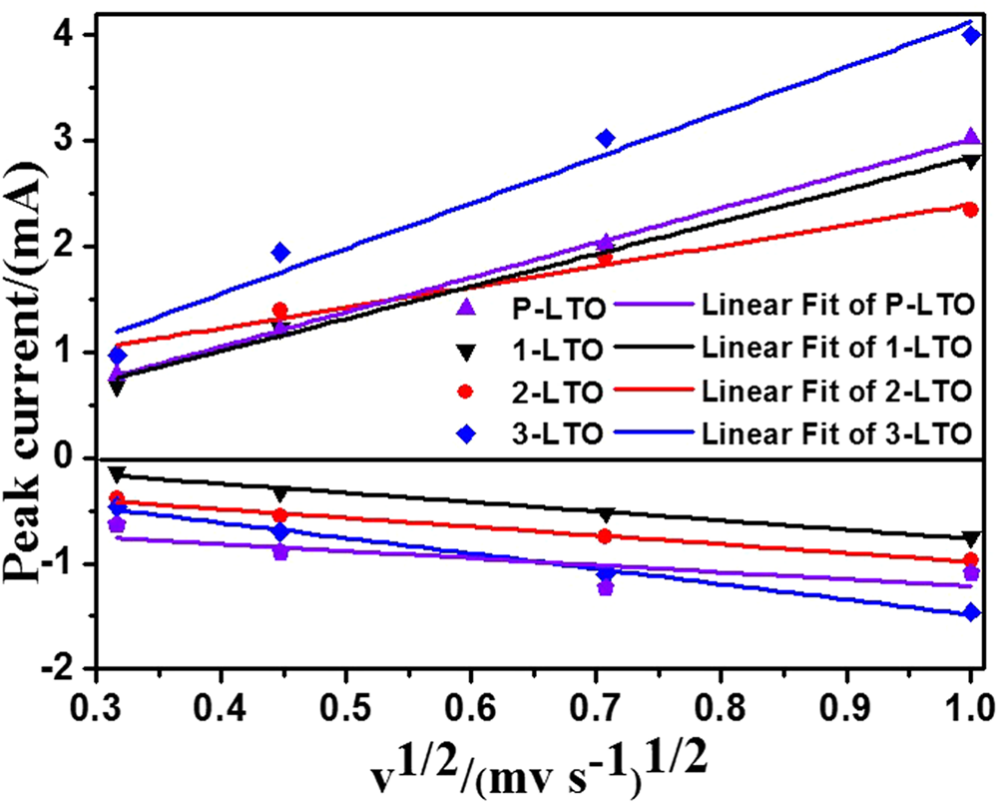
Relationship between the peak current (*i*_*p*_) and the square root of the scan rate (*v*^1/2^).

The relationship between the redox peak current (*i*_p_) and the square root of the scan rate (*v*^1/2^) is shown in Fig. 6. There is a linear relationship because the diffusion rate limits the process of lithium-ion insertion/extraction. For the semi-infinite and finite diffusion, the Randles-Sevcik equation was used to calculate the relationship between the redox peak current and the square root of the scan rate.

$${i}_{{\rm{p}}}=k{n}^{3/2}A{{D}_{{\rm{Li}}}}^{+1/2}{C}_{0}\,{v}^{1/2}$$

Here, *n* is the number of electrons in the redox reaction, *A* is the area of the electrode surface (cm^2^), *C*_0_ is the molar concentration of lithium ions (mol cm^−3^), *D*_Li_^+^ is the diffusion coefficient of lithium ions, and *v* is the scan rate of the potential (V s^−1^). As indicated in Table 1, 2-LTO has a larger diffusion coefficient than the other samples, which indicates that the presence of Ti^3+^ can effectively improve the lithium ion diffusion.

**Table 1:** Li^+^-ion diffusion coefficients of P-LTO, 1-LTO, 2-LTO, and 3-LTO electrodes calculated from the CV curves.

	P-LTO	1-LTO	2-LTO	3-LTO
*D*_oxidation_	7.3 × 10^−12^	1.7 × 10^−12^	9.3 × 10^−11^	5.6 × 10^−11^
*D*_reduction_	4.7 × 10^−13^	3.5 × 10^−12^	1.16 × 10^−11^	6.23 × 10^−12^

**Figure 7:**
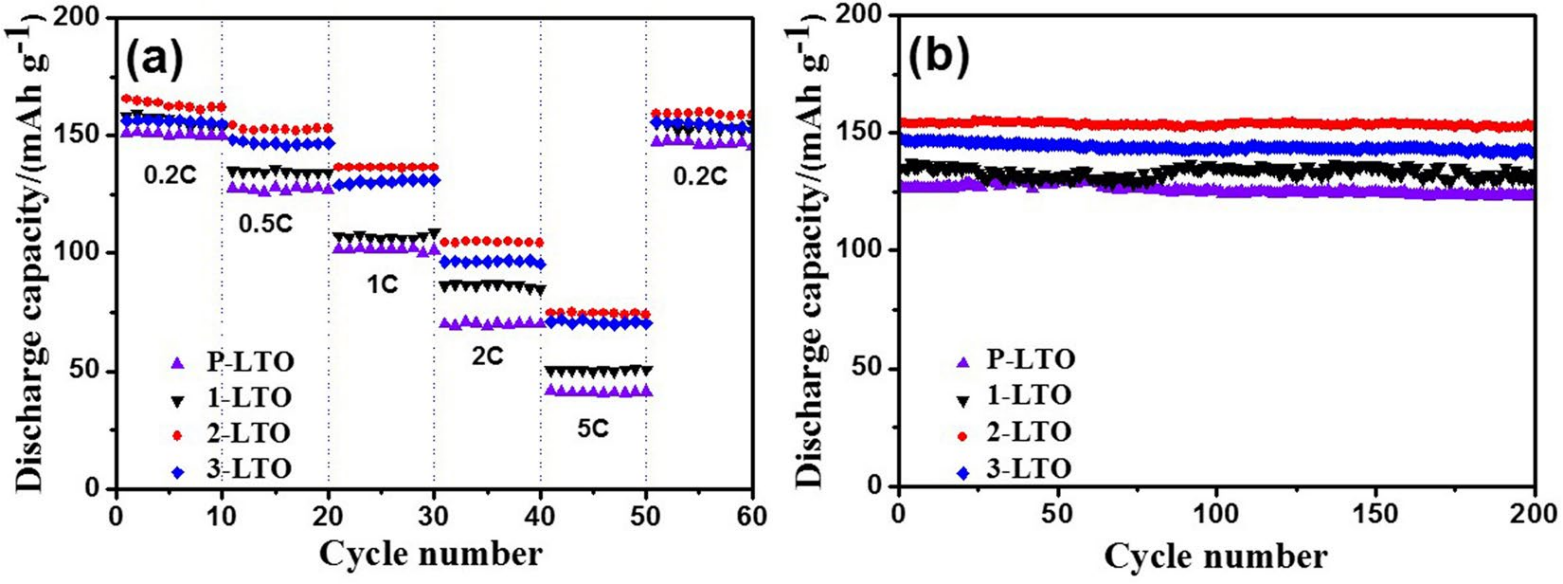
(**a**) The rate capability and (**b**) cycling performance of Li_4_Ti_5_O_12−*y*_ with different carbon black contents.

The cycling performance of the four samples at 0.5 C is displayed in Fig. 7a. The four samples have good cycling stability. The 2-LTO electrode showed an improved cycling performance compared with other electrodes. After an initial discharge capacity of 126.7, 135.4, 154.2, and 147.4 mA h g^−1^, the P-LTO, 1-LTO, 2-LTO, and 3-LTO samples achieved capacity retentions of 97.5%, 98.2%, 99.3%, and 96% after 200 cycles, respectively. Figure 7b compares the rate capability of the P-LTO, 1-LTO, 2-LTO, and 3-LTO samples at different current rates. The current density was increased gradually from 0.2 C to 5 C and returned to 0.2 C again. The cells were run for 10 cycles at each of the C rates. The P-LTO sample exhibited good discharge capacities and cycling stabilities at a low current rate of 0.2 C. However, its capacity dropped significantly with increasing current rate, whereas the samples with carbon additive exhibited relatively higher capacities. The discharge capacities of P-LTO are 127.3, 101.7, 70.1, 41.6, and 27.5 mA h g^−1^ at 0.2 C, 0.5 C, 1 C, 2 C and 5 C, respectively. The samples with carbon black additive exhibited better electrochemical performance than P-LTO. Among all samples, the 2-LTO sample exhibited the highest discharge capacity and best cycling stability, which is consistent with the results of CV measurements at 0.5 mV s^−1^ (Fig. SI 6). When the current density returned to 0.2 C, a stable capacity of 163.9 mA h g^−1^ was obtained without significant capacity fading. The excessive carbon black decreases the capacity of the 3-LTO sample. These results indicate that the presence of Ti^3+^ in Li_4_Ti_5_O_12_ can improve the rate capability, which agrees well with the previous results.

The linear scanning voltammetry (LSV) measurement was carried out to test the electronic conductivity of the samples. Figure SI 7 shows that the electrical conductivity of P-LTO, 1-LTO, 2-LTO, and 3-LTO samples were 1.1 × 10^−6^, 6.6 × 10^−5^, 2.2 × 10^−4^ and 1.1 × 10^−4^ S cm^−1^, respectively, which is higher than the pure Li_4_Ti_5_O_12_ (10^−9^ S cm^−1^) reported in the literature. This is mainly due to the ion radius of Ti^3+^ (0.67 Å) which is larger than that of Ti^4+^ (0.605 Å), and the increased radius of Ti^3+^ can change the lattice parameters of the Li_4_Ti_5_O_12_ material, making insertion and extraction of lithium ions easier, thus enhance the rate performance of Li_4_Ti_5_O_12_. To a certain extent, the electronic conductivity of Li_4_Ti_5_O_12_ increased with the increasing fraction of carbon black, when the amount of carbon black exceeded 2 wt. %, the electronic conductivity of the sample begins to decrease. It means excessive carbon exists in the sample in the form of impurities, which leads to bad electronic conductivity and poor electrochemical performance.

**Figure 8:**
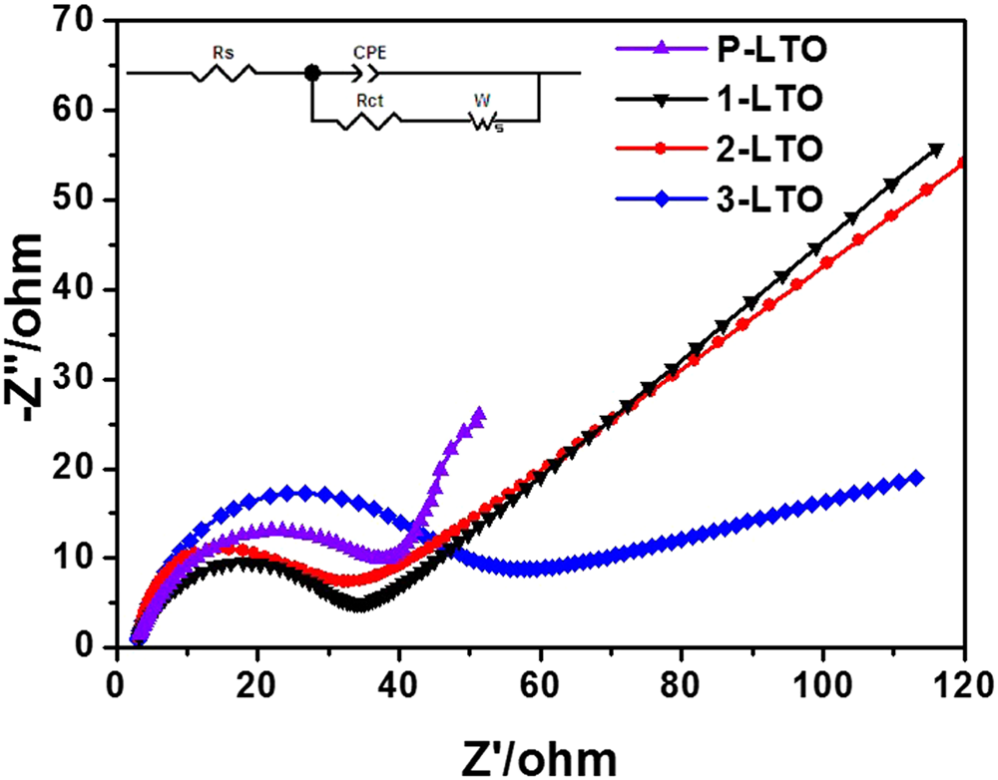
Impedance plots at room temperature for Li_4_Ti_5_O_12−*y*_ with different carbon black contents.

To investigate the differences in electrochemical performance, EIS measurements were carried out for the samples at a voltage of 1.55 V (vs. Li/Li^+^). The Nyquist plots for the cells are fitted to the equivalent circuit shown in Fig. 8. The EIS curves are composed of a depressed semicircle at the high- to intermediate-frequency range and a line in the low-frequency range. Usually, the charge transfer resistance (*R*_ct_) in the high middle-frequency region is related to lithium-ion charge transfer at the material interface, whereas the low-frequency Warburg region is related to the lithium-ion diffusion coefficients of the material. A higher exchange current density (*i*^0^ = *RT*/*n*F*R*_ct_) of the material results in improved high rate performance. The *R*_ct_ values of P-LTO, 1- LTO, 2-LTO, and 3-LTO are 38.8, 34.6, 31.7 and 54.8 Ω, respectively. Based on the above analysis, 2-LTO exhibited the best electrochemical characteristics and the lowest resistances, and, thus, the best cycling and rate performance.

## Conclusion

The formation mechanism of solid solution Li_4_Ti_5_O_12−*y*_ prepared by a one-step solid-state reduction reaction was investigated by *in situ* VT-XRD and TGA-DSC measurements. First, TiO_2_ loses some oxygen (O) and is converted to TiO_2−*x*_ under a reductive atmosphere. Then, Li_2_CO_3_ reacts with TiO_2−*x*_ (anatase) to form intermediate Li_2_TiO_3−*x*_. Li_2_TiO_3_ reacts with anatase in the presence of carbon black to form Li_4_Ti_5_O_12−*y*_ at 700 °C. Anatase starts to transform into rutile above 700 °C, and Li_2_TiO_3−*x*_ continues to react with TiO_2−*x*_ (rutile) and carbon black, generating Li_4_Ti_5_O_12−*y*_ at higher temperatures. In this method, carbon black served as a reductant and reduced the surface Ti^4+^ to Ti^3+^. The surface modification can improve the electrical conductivity and Li^+^ diffusion coefficient and improve rate capacity and cycling stability significantly. Many researchers have modified the surface of Li_4_Ti_5_O_12_ particles with carbon layers, which they claim can improve the electrical conductivity. However, most of them did not mention the changes in the contact surface between the carbon layer and the Li_4_Ti_5_O_12_ particles. Our findings reveal that the carbon layer can reduce the surface Ti^4+^ of Li_4_Ti_5_O_12_ particles to Ti^3+^ in the carbonization process, and to some extent can improve the electrical conductivity. We believe that these results provide a solid foundation for optimizing the synthetic method and further developing Li_4_Ti_5_O_12_ as an anode material for lithium-ion batteries.

## Supplementary information

Supplementary information
