# Supplementary material for: The formation mechanism of Li4Ti5O12−y solid solutions prepared by carbothermal reduction and the effect of Ti3+ on electrochemical performance
Source: Sci Rep. 2019 Mar 18;9:4774. doi: 10.1038/s41598-019-41206-0 (PMC6423044; doi:10.1038/s41598-019-41206-0)
Supplement: Supplementary file 1 — Supplementary information [file 41598_2019_41206_MOESM1_ESM.pdf]

Supplementary data

The formation mechanism of $Li_4Ti_5O_{12-y}$ solid solutions prepared by carbonthermal reduction and the effect of Ti^{3+} on electrochemical performance

Guijun Yang and Soo-Jin Park*

[*] Corresponding author: S. J. Park, Ph.D.

Department of Chemistry and Chemistry Engineering, Inha University, 253, Nam-gu, Incheon 402-751, Korea (south),

Tel.: +82-32-860-8438, Fax: +82-32-860-8438

E-mail: sjpark@inha.ac.kr.

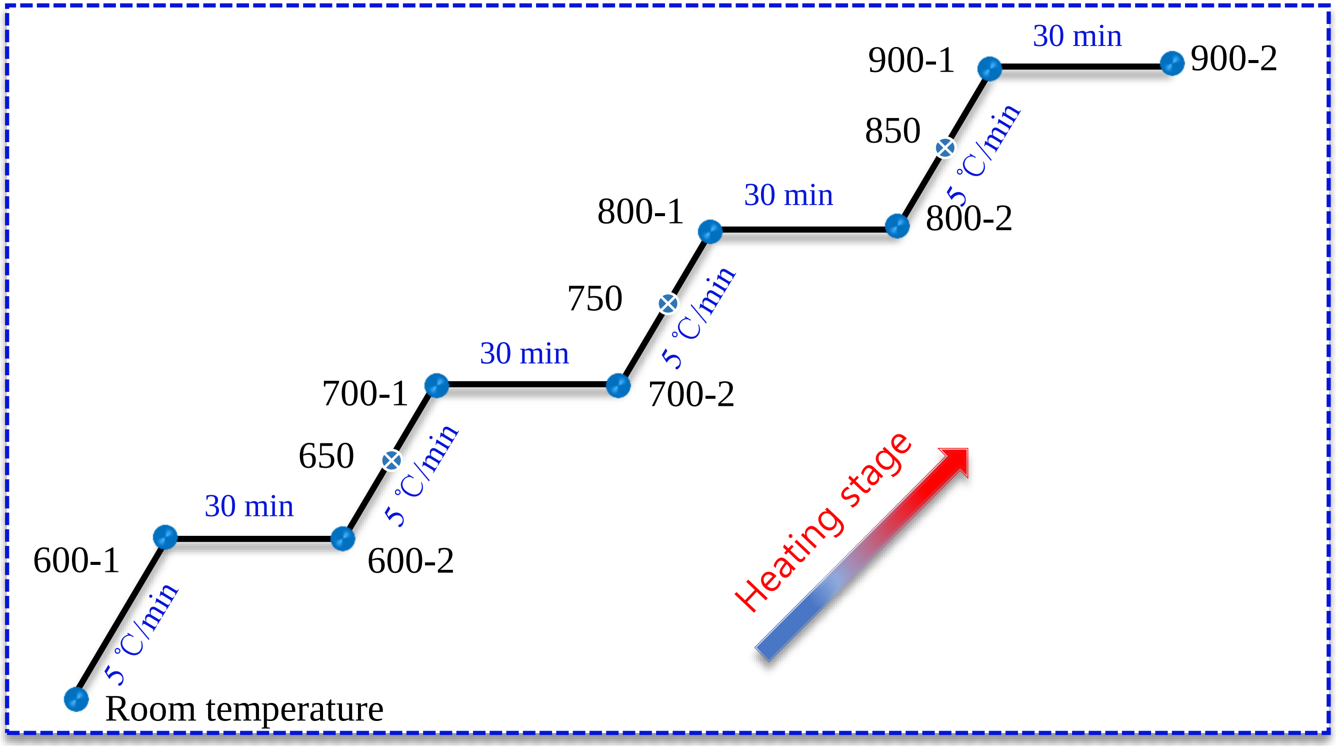

Fig. SI 1. Schematic of *in situ* VT-XRD measurement conditions from room temperature to 900 ℃.

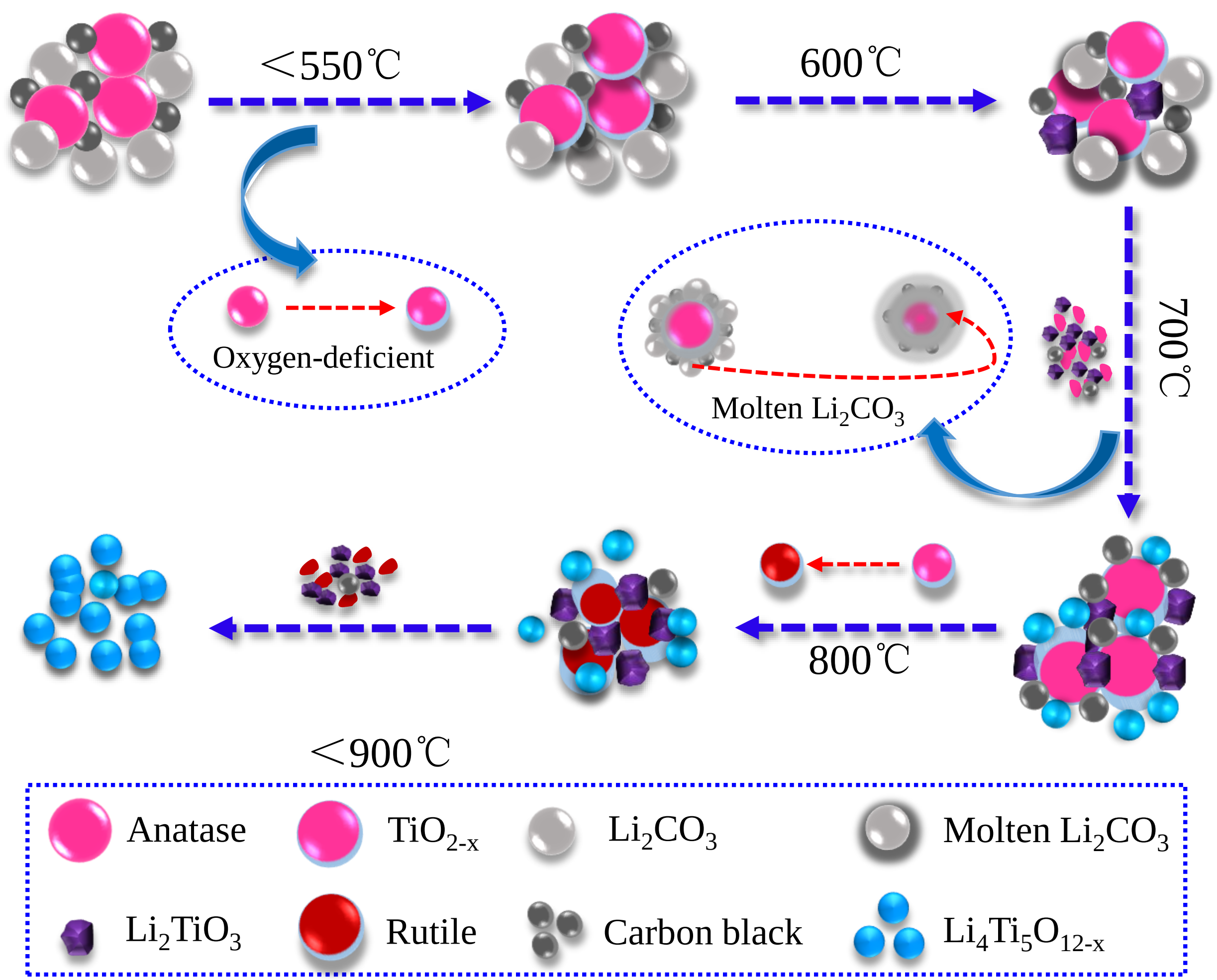

Fig. SI 2. Schematic of the formation mechanism of solid solution $Li_4Ti_5O_{12-x}$.

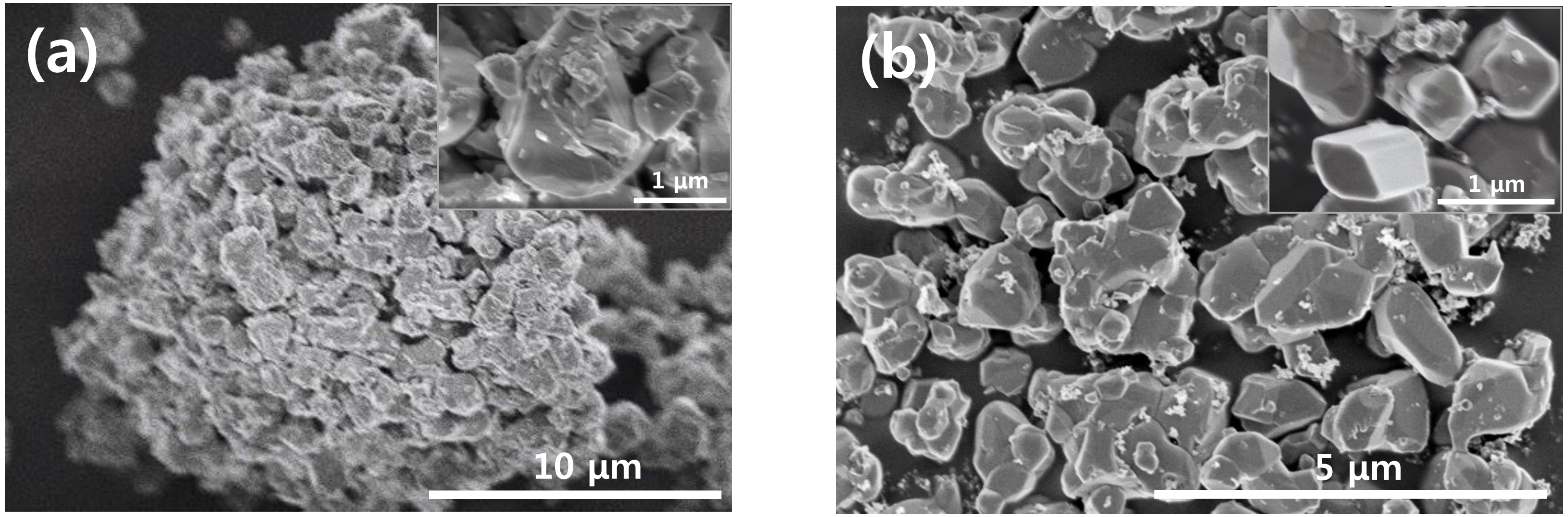

Fig. SI 3. SEM images of (a) P-LTO and (b) 2-LTO.

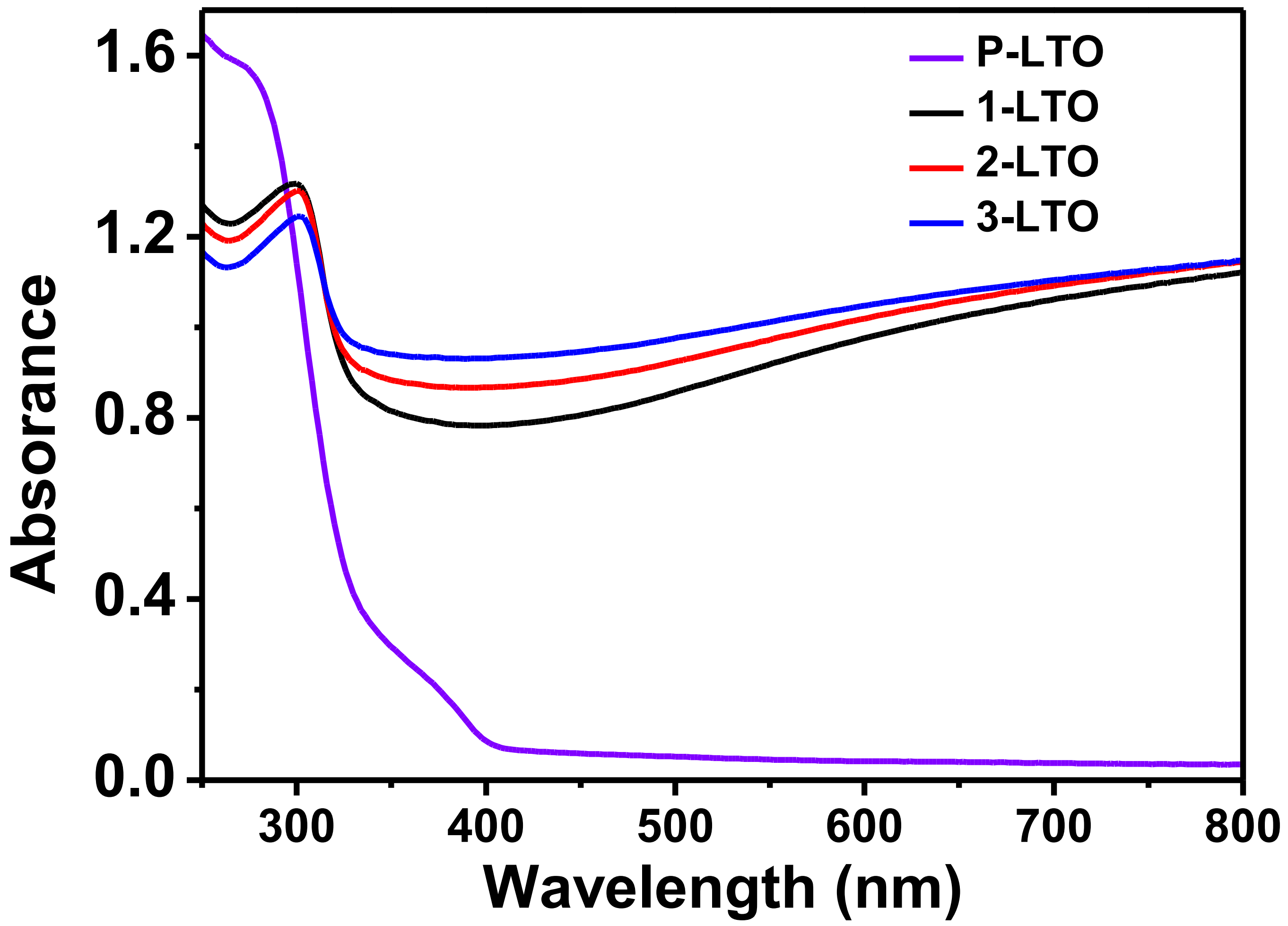

Fig. SI 4. UV-vis diffraction spectra of P-LTO, 1-LTO, 2-LTO and 3-LTO.

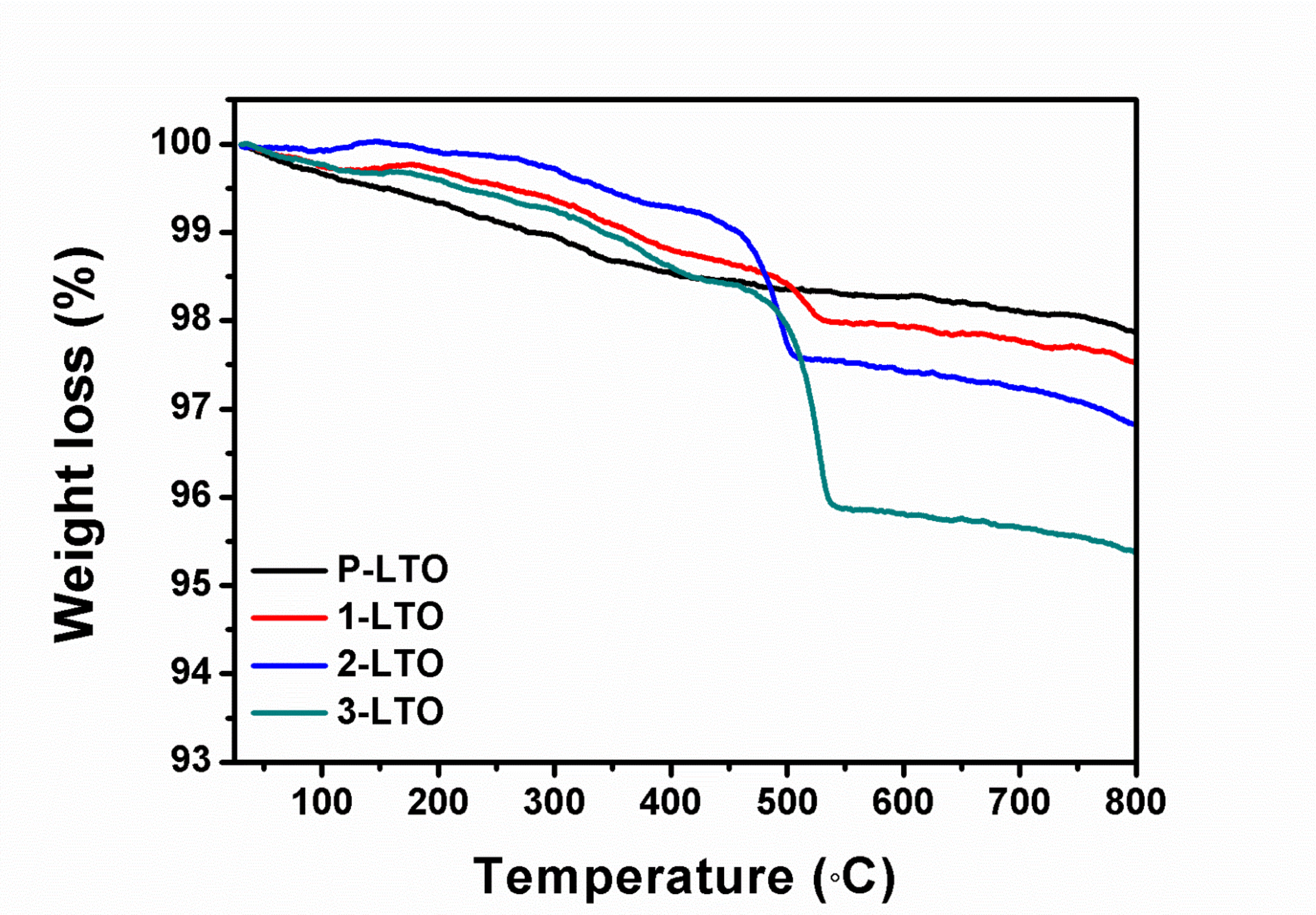

Fig. SI 5. TGA curves of P-LTO, 1-LTO, 2-LTO and 3-LTO samples from room temperature to 800 ℃ under air atmosphere.

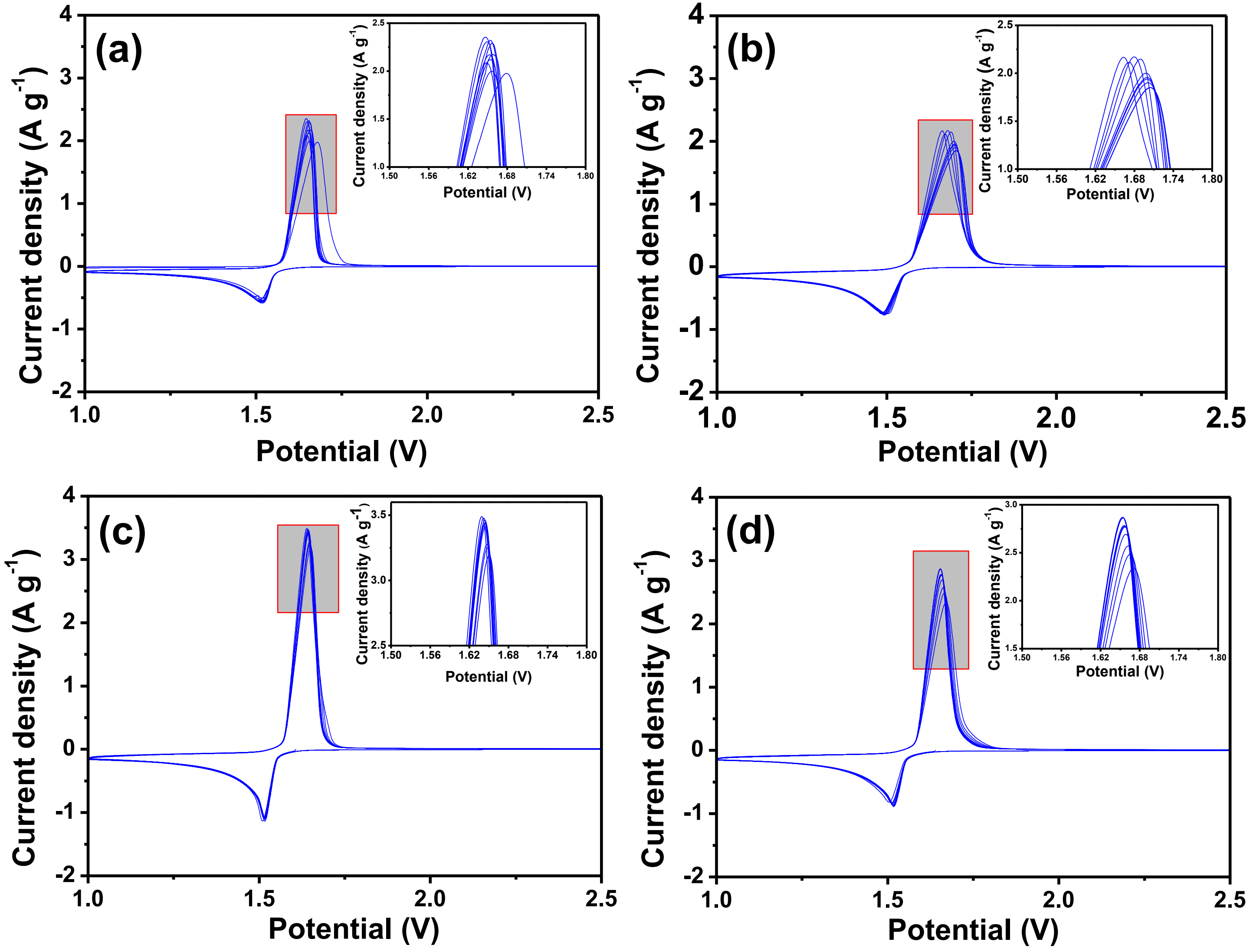

Fig. SI 6. Cyclic voltammogram properties of (a) P-LTO (b) 1-LTO (c) 2-LTO (d) 3-LTO at 0.5 mV s^{-1}.

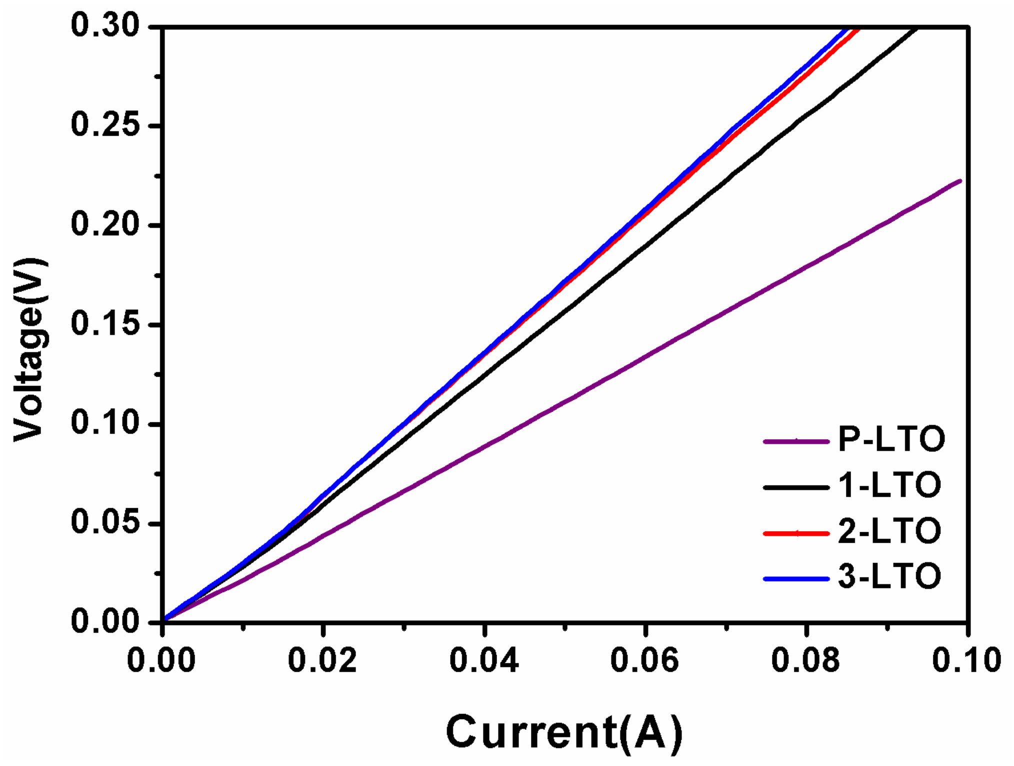

Fig. SI 7. The electronic conductivity of P-LTO, 1-LTO, 2-LTO and 3-LTO.
